# The Real "Catch" in Clinical Automation: Rethinking Artificial Intelligence for Healthcare Workflows

**DOI:** 10.7759/cureus.111390

**Published:** 2026-06-23

**Authors:** B R Sathyakrishna, Andrew Lourdunathan, Punith Nagarajappa, Izhar Vinson Iyapillai, Ravichandran Subramaniam

**Affiliations:** 1 General Surgery, St. Martha's Hospital, Bengaluru, IND; 2 Neurosurgery, Gleneagles Hospital, Chennai, IND; 3 General Surgery, SRM Medical College Hospital & Research Centre, Chennai, IND

**Keywords:** alert fatigue, artificial intelligence, clinical automation, clinical decision support systems, electronic health records, healthcare workflows, medical informatics, patient safety, smart on fhir

## Abstract

Artificial intelligence (AI) has demonstrated impressive performance in controlled retrospective studies, yet many systems struggle to achieve meaningful operational utility when deployed in real-world clinical environments. Live healthcare workflows are characterized by documentation burdens, user-interface friction, variable data quality, and competing demands on clinician attention. These challenges can reduce the effectiveness of predictive systems and contribute to alert fatigue, workflow inefficiencies, and administrative burdens. This editorial, presented as a narrative perspective and conceptual synthesis, argues that successful clinical AI implementation requires a shift in focus from theoretical predictive accuracy toward practical workflow integration and floor efficiency. Effective clinical systems should function as supportive layers within healthcare workflows by reducing cognitive burden, improving documentation quality, and facilitating safer clinical decision-making. Approaches such as tiered information presentation, workflow-aware design, human oversight, and strict medicolegal frameworks, including mandatory electronic health record (EHR) "watermarking" and continuous audit trails, may help improve adoption while minimizing unintended consequences. By supporting accurate documentation and efficient chart completion, integrated AI has the potential to enhance operational performance, preserve clinician cognition, and facilitate safer patient care.

## Editorial

The laboratory vs. the ward

The rapid advancement of artificial intelligence (AI) in healthcare has created a paradigm where predictive models routinely achieve exceptional accuracy in controlled, retrospective environments. Yet, a well-documented chasm exists between algorithmic performance in the laboratory and actual clinical utility at the bedside. When high-performing models are deployed into live electronic health record (EHR) systems, they frequently encounter a harsh operational reality: clinical data is notoriously imperfect, and clinical attention is a strictly finite resource. The fundamental problem is that many AI tools treat the EHR as an infallible source of ground truth and the clinician as an infinitely available processor of alerts.

In reality, the point of care, whether during a chaotic ward round or a fast-paced surgical consultation, is defined by friction. As established by Meeks et al. [1] in their foundational sociotechnical model, EHR-related safety concerns rarely stem from isolated software bugs; rather, they arise from the complex interplay between technical interfaces, hidden dependencies, and the human realities of clinical workflows. When an AI model assumes flawless data entry, it becomes highly vulnerable to routine drop-down menu fatigue or copy-paste documentation errors, essentially adopting human mistakes as empirical fact. Consequently, this "garbage in, garbage out" pipeline transforms what should be a supportive clinical co-pilot into a source of cognitive overload.

Stabilizing core systemic integrity is an absolute prerequisite to algorithmic integration. AI remains operationally ineffective if layered over inherently flawed workflows. Optimizing hospital performance requires a framework wherein floor efficiency -- defined here as the seamless optimization of ward throughput, timely documentation completion, and the preservation of clinician time -- rigorous time management, and continuous technological innovation drive sustainable healthcare delivery. However, successful implementation relies heavily on the integrity of the human systems operating it; deploying workflow-level corrective systems to address procedural errors must take precedence over advanced automation.

Floor efficiency and AI reliability are closely interconnected. In high-volume wards, fragmented workflows force clinicians into rapid documentation behaviors that degrade data quality and increase cognitive fatigue. As floor inefficiency worsens, predictive systems become increasingly dependent on incomplete, delayed, or inaccurate inputs. Consequently, many apparent "AI integration failures" may actually represent operational failures occurring upstream at the level of clinical workflow design. Therefore, we argue that clinical AI must be evaluated not only by its retrospective predictive accuracy, but by its practical ability to improve workflow, reduce documentation burdens, and support clinician judgment.

The "garbage in" problem and UI bottlenecks

The modern EHR is often optimized for billing and compliance rather than clinical utility. Under severe time constraints to meet rigorous accreditation standards like the National Accreditation Board for Hospitals and Healthcare Providers (NABH), structured data entry frequently suffers from "drop-down fatigue." For example, a clinician prescribing postoperative orders might accidentally select an intravenous (IV) route instead of per-oral (PO) simply because they are adjacent in a densely packed list. The key lesson here is that predictive AI models cannot distinguish these mechanical UI errors from deliberate clinical interventions, leading to skewed algorithmic outputs. To build a resilient clinical minimum viable product (MVP), conceptualized in this context as a foundational, workflow-integrated support tool rather than a fully autonomous diagnostic engine, the AI must serve as a defensive layer. This defensive layer acts as an intermediary system that intercepts and queries potential data entry errors before they reach the predictive layer.

Compounding this poor data fidelity is the historic tuning of clinical decision support systems (CDSS) for high sensitivity, generating an unsustainable volume of low-priority warnings. Studies have reported alert override rates exceeding 90% in some clinical environments, reflecting the substantial burden imposed by low-priority warnings [2]. Furthermore, when an AI is highly accurate, "automation bias" presents a distinct danger. The recent global surge in the adoption of generative AI and large language models (LLMs) in clinical settings makes this risk even more urgent. A fatigued surgeon at the end of a 24-hour call will be tempted to click "Approve" on preemptively staged classifications without validation, risking inattentive approval behaviors. To build a resilient clinical MVP, developers must move away from interruptive pop-ups and engineer "friction-by-design" for critical interventions, ensuring clinicians remain active cognitive participants.

Scaling national frameworks and redefining ROI

The long-term success of large-scale public health insurance programs, such as India’s Ayushman Bharat Pradhan Mantri Jan Arogya Yojana (AB-PMJAY), relies heavily on efficient claims processing [3]. However, immense insurance paperwork burdens and high claim rejection rates stem from non-standardized clinical documentation. Public healthcare audit data reveal a massive outstanding recovery deficit under AB-PMJAY because clinical documentation processing is severely delayed when high-volume wards discharge patients before manual paperwork is completed at centralized administrative counters [4]. Rapid bed turnaround goals directly compromise the meticulous data capture required by bundled-pricing reimbursement frameworks [3].

As noted by Joseph [5], AI health interventions in developing nations often fail to scale -- remaining trapped in "pilotitis" -- due to a lack of foundational interoperability and systemic readiness. To achieve "Gold" quality certification under AB-PMJAY, hospitals must maintain full NABH accreditation, demanding standardized medical records. The necessity of this standardization is twofold. First, high-quality documentation is vital for patient care and clinical accountability; operative notes must align with rigorous benchmarks, such as the Royal College of Surgeons (RCS) guidelines, which mandate explicit details on preoperative diagnosis, operative findings, implants used, and postoperative plans. Second, this documentation directly dictates coding, claims, and institutional reimbursement. When fatigued surgeons omit details, it triggers a cascade of administrative failures: International Classification of Diseases, 10th Revision (ICD-10) coding errors, AB-PMJAY claim rejections, and delayed reimbursements, driving institutional "participation fatigue". In this context, an AI MVP transitions to a systemic enabler. By functioning as an ambient, defensive layer within the EHR, the AI can actively audit a surgical note against both RCS and NABH metrics in real-time. Instead of relying on counter-based compliance, this ambient "pull" dynamic intercepts data gaps directly on the ward, instantly prompting the user before a note is finalized. This removes the workflow lag driving institutional recovery deficits, mapping charts seamlessly to corresponding health benefit packages.

Thus, the true return on investment (ROI) of a clinical AI MVP is found not in its area under the curve (AUC), but in its direct impact on floor efficiency and ward throughput. In high-volume wards, efficiency is dictated by standard operating procedures (SOPs) and rapid bed turnover. An integrated AI should parse dictated notes, cross-reference them against hospital postop SOPs, and preemptively stage the correct ICD classifications and discharge criteria for final approval. By potentially mitigating the administrative bottleneck of chart closure, the AI may help accelerate safe patient discharge, transforming into the operational engine of the surgical ward.

Policy frameworks, medicolegal traps, and guarding cognition

The foundational principle of a clinical MVP is human-in-the-loop (HITL) design. However, deploying AI in live clinical environments introduces profound medicolegal friction: the liability of possessing a predictive insight without acting upon it. To navigate this, emerging policy frameworks must explicitly classify clinical AI software as "high-risk." This classification necessitates transparent data governance and mandatory declarations at every stage of patient care. Hospitals must treat silent deployments under the same ethical frameworks as clinical trials, utilizing them for operational metrics rather than high-acuity diagnostics, backed by legally binding data-processing agreements that prevent vendors from using ward data to train external commercial models.

Protecting clinical integrity requires strict attention to provenance to avoid "algorithmic hallucinations" from becoming codified as medical fact. Institutions must mandate that every AI-generated output, whether a drafted operative note, a staged ICD code, or a final discharge summary, is visibly "watermarked" within the EHR. This step-by-step declaration must include a timestamp, the underlying data points used, and the authorizing clinician's signature. This continuous audit trail does more than establish liability; it serves as a critical surveillance mechanism to detect algorithmic drift, clearly indicating to developers and administrators exactly when an underlying AI model requires retraining. In India, frameworks under the Ayushman Bharat Digital Mission (ABDM) are pushing for clear data governance.

Ultimately, guarding cognitive capacity means recognizing that human working memory is finite; AI must pivot toward intelligent "pull" dynamics. If an AI detects a non-critical downward trend in hemoglobin, it should not interrupt the surgeon during active postoperative data entry for another patient. It should silently stage that insight until the physician opens that specific patient's chart, acting as "ambient intelligence" -- an unobtrusive background system that passively monitors data and surfaces insights only when contextually relevant -- that utilizes pre-attentive visual attributes to allow a clinician to grasp a trajectory in a fraction of a second.

Limitations

We explicitly acknowledge that this perspective is a narrative synthesis and conceptual perspective drawn directly from the operational realities of high-volume surgical wards, rather than a quantitative, randomized trial of alert override rates. The primary purpose of this manuscript is to provoke a necessary paradigm shift in how medical informatics approaches clinical design. Furthermore, regulatory frameworks governing AI, such as the European Union AI Act and the ABDM, are in active legislative evolution. Consequently, the medico-legal landscape for "shadow mode" deployments will require continuous re-evaluation in the coming years. The models described cannot account for the full spectrum of unique clinical scenarios, emerging ethical considerations, or the ultimate element of individual human free will and judgment. The concepts discussed are theoretical and intended to stimulate discussion regarding workflow-centered AI design in healthcare environments. Final clinical responsibility must remain with the treating clinician and the institutional governance framework.

To formally investigate the empirical data behind these operational workflows and socio-technical tradeoffs, a comprehensive systematic review has been initiated. The formal protocol for this secondary research is registered in the International Prospective Register of Systematic Reviews (PROSPERO ID: CRD420261405820), which will explicitly explore the quantitative and qualitative outcomes of AI documentation systems in inpatient surgical settings.

Finally, the architectural concepts and design philosophies discussed in this manuscript are purely theoretical and advisory. They are not intended to constitute medical, legal, or operational advice.

Conclusions

The failure of many clinical AI systems does not primarily reflect inadequate algorithms, but rather the operational instability of the environments into which they are deployed. High-volume healthcare systems are constrained by fragmented workflows, documentation fatigue, administrative overload, and limited clinician attention. Under such conditions, even highly accurate predictive models become vulnerable to unreliable inputs and unsafe implementation patterns.

Successful clinical AI integration, therefore, depends on strengthening floor efficiency as much as improving algorithmic performance. Systems designed around ambient support, human-in-the-loop validation, tiered salience, and workflow-aware implementation are more likely to reduce cognitive burden while improving documentation quality and patient throughput. The true value of clinical AI lies not simply in predictive accuracy metrics but in its ability to stabilize operational workflows, preserve clinician cognition, and support safer, more efficient care delivery. To transition from theoretical implementation to measurable success, hospitals deploying clinical AI must establish rigorous baseline metrics. Future empirical evaluations should prioritize tracking documentation time, alert override rates, chart closure times, discharge turnaround, coding accuracy, claim rejection rates, overall clinician workload, and the incidence of patient safety events.

Protocol registration note

The conceptual framework presented in this editorial establishes the operational rationale for a broader, empirical investigation. The quantitative and qualitative "what" behind these automation tradeoffs is currently being examined via an ongoing study titled "The Trade-off Between Administrative Efficiency and Clinical Vigilance: A Systematic Review of AI Documentation Systems in Surgical Workflows," which has been formally registered with PROSPERO (Registration Number: CRD420261405820).
